# Restricted Social Engagement Is Associated With Depressive Symptoms in Older Chinese Adults: A Nationwide Cross‐Sectional Study

**DOI:** 10.1002/hsr2.71262

**Published:** 2025-09-21

**Authors:** Zheng Guo, Yulu Zheng, Yahong Zhu, Huacheng Wang, Jian Geng, Hengrui Liu, Piyong Ma, Lois Balmer, Xingang Li

**Affiliations:** ^1^ School of Medical and Health Sciences Edith Cowan University Perth Australia; ^2^ Centre for Precision Health Edith Cowan University Perth Australia; ^3^ School of Science Edith Cowan University Perth Australia; ^4^ Intensive Care Unit The Second Hospital of Tianjin Medical University Tianjin China; ^5^ Department of Clinical Laboratory Tai'an City Public Health Medical Center Tai'an China; ^6^ Department of Biochemistry, Hopkins Building University of Cambridge Cambridge UK; ^7^ Critical Care Medicine China‐Japan Union Hospital of Jilin University Changchun China; ^8^ Nutrition and Health Innovation Research Institute Edith Cowan University Perth Australia

**Keywords:** COVID‐19, depressive symptom, elderly, social activity

## Abstract

**Background and Aims:**

Social isolation has been linked to depressive symptoms in older adults. Previous studies have shown that greater engagement in social activities is associated with better mental health. However, it remains unclear whether restricted social engagement is related to poorer mental health outcomes. This study examined the association between reduced social activities/networking and the presence of depressive symptoms among community‐dwelling older Chinese adults.

**Methods:**

During the COVID‐19 pandemic, people chose to minimize going out to reduce the risk of infection, creating a natural setting that mimicked a passive intervention of restricted social engagement for community‐based individuals. Data were drawn from 7288 participants aged ≥ 65 years from the fifth wave (2019–2020) wave of the China Health and Retirement Longitudinal Study. Depressive symptoms were measured using the 10‐item Center for Epidemiologic Studies Depression Scale. Social activities/networking changes due to COVID‐19 were self‐reported. Multivariable logistic regression examined associations between activity changes and depressive symptoms, adjusting for demographics and anxiety/fear levels.

**Results:**

Among 7288 participants (49.8% female;mean age 73.1, SD = 6.6), 41.4% exhibited depressive symptoms. The prevalence of depressive symptoms was higher among females (50.3%) than males (32.7%). Higher education levels were associated with a lower prevalence of depressive symptoms. Participants reporting anxiety or fear were more likely to exhibit depressive symptoms. After adjusting for potential confounders, including social demographics and other mental problems, multivariate logistic regression analysis revealed reduced contact with other people was a significant risk factor for depression, whereas reduced participation in social gatherings was unexpectedly associated with a lower risk of depressive symptoms.

**Conclusions:**

Reduced social engagement and related health behaviors were associated with an increased likelihood of depressive symptoms among Chinese older adults during the COVID‐19 pandemic. Interventions that promote social participation and healthy lifestyles may help protect mental health in this vulnerable population.

## 1 Introduction

Depression remains the leading contributor to the global burden of mental health–related disease, accounting for more than 56.3 million years lived with disability (YLDs) and affecting approximately 332 million people in 2021 [1]. Untreated, depression can have severe consequences, including other illnesses [2] and even suicide [3]. The prevalence of elevated depressive symptoms persisted from 27.8% in 2020 (95% CI: 24.9, 30.9) to 32.8% in 2021 (95% CI: 29.1, 36.8) during the COVID‐19 pandemic [4].

Prior studies have established that social participation and networks are beneficial for mental well‐being, potentially reducing risks of anxiety, cognitive decline, and depression in older adults [5, 6, 7]. Longitudinal evidence also underscores the negative effects of prolonged social isolation, which can lead to diminished psychological well‐being, cognitive function, and decreased life satisfaction in the elderly [8, 9, 10]. Physical activity, closely tied to social participation, has likewise been shown to enhance health outcomes and reduce risks of depression, functional decline, and mortality [11, 12, 13].

The COVID‐19 pandemic has had widespread impacts on social, occupational, and economic aspects of daily life [14, 15]. To mitigate infection spread and flatten the pandemic curve, however, many countries' governments adopted lockdowns and a series of restrictions including bans on public gatherings, social distancing measures, and stay‐at‐home policies. For instance, government policies required individuals to contact only a limited number of people outside their household [16]. COVID‐19 test‐positive individuals were required to have a 2‐week or longer self‐isolation or quarantine. Moreover, individuals were mandated to practice social distancing and abstain from non‐essential activities [17]. Consequently, people spent a greater part of their daily lives at home with reduced interpersonal interaction [18], engaging in homeschooling [19] and remote work [20]. While these measures effectively curbed the spread of the infectious disease, they drastically constrained social activities, and interpersonal networking.

These social and behavioral restrictions could have unintended negative impacts on people's mental health [21], with loneliness emerging as a key factor strongly linked to depression among older adults [22]. The findings of Kim and Park [9] highlight that such prolonged social isolation, as experienced during pandemics, can exacerbate mental health challenges by weakening stress‐coping mechanisms and reducing life satisfaction and cognitive decline, particularly in older populations [8, 9, 10]. In addition, studies have indicated that loneliness, isolation, and depression are all predictive of poorer disease outcomes in older populations. Meanwhile, mobility restrictions and physical distancing measures have led to reduced access to essential care for this demographic [22]. Notably, however, the existing evidence remains limited regarding how changes in social engagement correlate with depressive symptoms among older adults during the pandemic.

As one of the first countries impacted by COVID‐19, China instituted stringent lockdown and social distancing measures relatively early in the pandemic timeline. This created a unique natural experiment to examine how forced changes in social engagement and activities related to mental health outcomes in older Chinese adults. Using nationally representative data from the China Health and Retirement Longitudinal Study (CHARLS), the current study aimed to evaluate associations between changes in social activities/networking due to COVID‐19 and depressive symptom levels in a sample of Chinese adults aged 65 and older. Identifying specific social/behavioral factors linked to depression can inform public health efforts to support the mental wellbeing of older populations facing ongoing or future public health emergencies.

## 2 Methods

### 2.1 Patient and Public Involvement

In the current study, the data were derived from the fifth wave of the China Health and Retirement Longitudinal Study (CHARLS) [23] which was conducted between 2019 and 2020 with 19,395 respondents. Administered by the National School of Development (China Center for Economic Research) at Peking University, the CHARLS is a nationally representative community‐based population survey in China.

The survey was designed to gather data from Chinese individuals and families for multidisciplinary research in economics, sociology, and demography. It focused on adults aged 45 and above along with their spouses, representing both urban and rural households in China. Approved by the institutional review board of Peking University, the CHARLS survey used a multi‐stage cluster sampling design across 30 provinces. The participants' selection followed a three‐level sampling framework: county/city, village/neighborhood committee, and household. Using probability proportional to size (PPS), 150 counties/cities were first chosen. In each selected county/city, three village or neighborhood committees were sampled, and subsequently, 80 households per committee were randomly selected via a specialized Geographic Information Systems program. From each household, one adult aged 45 or older and their spouse were randomly chosen to participate in face‐to‐face interviews for data collection. The CHARLS data set, accessible through its official website (charls.ccer.edu.cn/en) upon approval, ensures national representativeness for community‐based population research.

**Figure 1:**
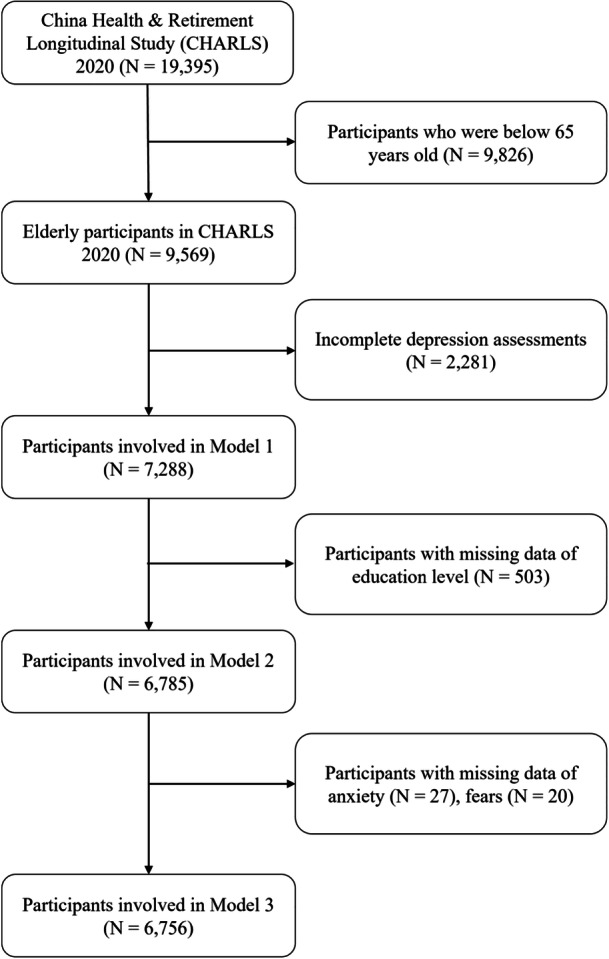
Flowchart of the study design.

In accordance with the objectives of the present study, exclusion criteria for the study participants were established as follows: (1) individuals younger than 65 years of age; (2) those lacking data from the 10‐item Center for Epidemiologic Studies Depression (CESD‐10); (3) participants without social activity and networking questionnaire data; and (4) individuals missing anxiety and fears data. A total of 9569 participants aged 65 years and older were included in the analysis. Among these, 7288 individuals underwent both social activity and networking questionnaires and depressive symptoms assessment (Figure 1).

### 2.2 Depressive Symptoms Assessment

The 10‐item Center for Epidemiologic Studies Depression (CESD) scale was utilized to assess depressive symptoms in the fifth‐wave survey [24]. Developed by adapting the original version of the 20‐item CESD [25], this version excluded highly redundant items. Respondents were asked to rate “how often you felt during the past week” for each item on a 4‐point Likert scale: 0 (rarely or none of the time; < 1 day), 1 (some of the time; 1 ~ 2 days), 2 (much or a moderate amount of the time; 3 ~ 4 days), or 3 (most or all of the time; 5 ~ 7 days). Total score ranges from 0 to 30, with lower scores indicating fewer depressive symptoms. Previous research has established a cut‐off score of 10 as having reasonable sensitivity and specificity for identifying depression in Chinese older adults [24]. In the current study, participants with CESD‐10 score ≥ 10 were classified into the depressive symptoms (DS) group, while those with scores < 10 were assigned to the no depressive symptoms (NDS) group [26].

### 2.3 Assessment of Social Activities and Networking

Based on the CHARLS questionnaire survey, social activities and networking were defined as the individual's participating in various activities, including interaction with friends (e.g., phone calls, text messages, home visits), engagement in hobby groups (e.g., Mahjong, chess, cards games), and involvement in sports (encompassing vigorous, moderate and light physical activities) [27]. In the current, we aim to explore the association between social activities and networking during COVID‐19 and depressive symptoms. Therefore, the respondents were asked, “Compared with before COVID‐19, has the time/amount/frequency of these social activities or networking increased/decreased/unchanged”.

### 2.4 Covariates

The covariates included age, sex, marital status, education level which were obtained through CHARLS questionnaires. In this study, marital status was divided into three groups: married, divorced/separated/widowed, and never married. Education level was divided into four groups: primary school or below, middle school, high school or vocational school, college degree or above. Anxiety and fears during COVID‐19 were self‐reported by participants.

### 2.5 Statical Analysis

In this study, all the data were obtained from the CHARLS database. Descriptive statistical methods were employed to analyze the characteristics of the study subjects. Continuous variables were presented as mean (M) ± standard deviation (SD), while categorical variables were expressed as n (%). Students' *T* test or *χ* ^2^ test was used to compare continuous and categorical variables, respectively. Taking depressive symptoms as the dependent variable and each type of social activity and networking as independent variables, multivariate logistic regression was performed to evaluate the association between depressive symptoms and social activities/networking among the elderly during COVID‐19. Missing data was minimal, constituting less than 1% of the total sample size. To ensure the robustness of the analysis, participants with missing values were excluded using listwise deletion. All analyses were conducted using R software (version 4.3.1 R Foundation), with statistical significance defined as two‐tailed *p* < 0.05.

## 3 Results

### 3.1 Characteristics of the Study Population

The characteristics of the study participants are presented Table 1. Among the 7288 participants included in the current study, all of whom met the inclusion criteria, 3661 (50.2%) were males, and the mean (SD) age was 73.1 (6.6). The detailed selection process for participants is illustrated in the flowchart (Figure 1). It was observed that 41.4% of the elderly exhibited depressive symptoms. Sex (*p* < 0.001), educational level (*p* < 0.001), marital status (*p* < 0.001), anxiety (*p* < 0.001), fears (*p* < 0.001), times of going outdoors (*p* < 0.001), time spent outdoors (*p* < 0.001), time of moderate activities (*p* = 0.02), time of light activities *(p* = 0.048), frequency of visiting others (*p* < 0.001), frequency of playing mahjong, chess, and cards (*p* < 0.001), frequency of dancing outdoors (*p* = 0.03), frequency of calling and messaging (*p* < 0.001), smoking (*p* < 0.001), drinking (*p* < 0.001), sleeping duration (*p* < 0.001), and food intake (*p* < 0.001) were found to be significantly different between participants with and without depressive symptoms (DS).

**Table 1:** Comparison of sample characteristics between DS and NDS in the elderly.

Characteristics		Overall (*N* = 7288)	NDS (*N* = 4268)	DS (*N* = 3020)	*p*‐value
Age (mean ± SD)		73.1 (6.6)	72.0 (5.8)	72.1 (5.66)	0.202
Sex *N* (%)	Male	3661	2465 (57.8%)	1196 (39.6%)	< 0.001
	Female	3627	1803 (42.2%)	1824 (60.4%)	
Education level *N* (%)	Primary school or below	5102	2753 (69.9%)	2349 (82.5%)	< 0.001
	Middle school	1089	736 (18.7%)	353 (12.4%)	
	High school or Vocational school	497	369 (9.4%)	128 (4.5%)	
	College degree or above	97	80 (2.0%)	17 (0.6%)	
Marital status *N* (%)	Married	5683	3484 (81.6%)	2199 (72.8%)	< 0.001
	Divorce/separated/widowed	1562	759 (17.8%)	803 (26.6%)	
	Never married	43	25 (0.6%)	18 (0.6%)	
Anxiety	Yes	4831	3171 (74.5%)	1660 (55.3%)	< 0.001
	No	2430	1087 (25.5%)	1343 (44.7%)	
Fears	Yes	4692	3040 (71.2%)	1652 (54.7%)	< 0.001
	No	2576	1219 (28.6%)	1357 (44.9%)	
Times of going outdoors	Increased	50	27 (0.6%)	23 (0.8%)	< 0.001
	Not Changed	2918	1593 (37.3%)	1325 (43.9%)	
	Decreased	4320	2648 (62.0%)	1672 (55.4%)	
Time spent outdoors	Increased	57	33 (0.8%)	24 (0.8%)	< 0.001
	Not Changed	2957	1585 (37.1%)	1372 (45.4%)	
	Decreased	4274	2650 (62.1%)	1624 (53.8%)	
Time of intense activities	Increased	73	36 (0.8%)	37 (1.2%)	0.065
	Not Changed	5522	3269 (76.6%)	2253 (74.6%)	
	Decreased	1693	963 (22.6%)	730 (24.2%)	
Time of moderate activities	Increased	229	121 (2.8%)	108 (3.6%)	0.02
	Not Changed	5424	3149 (73.8%)	2275 (75.3%)	
	Decreased	1635	998 (23.4%)	637 (21.1%)	
Time of light activities	Increased	155	88 (2.1%)	67 (2.2%)	0.048
	Not Changed	3942	2260 (53.0%)	1682 (55.7%)	
	Decreased	3191	1920 (45.0%)	1271 (42.1%)	
Frequency of visiting others	Increased	29	15 (0.4%)	14 (0.5%)	< 0.001
	Not Changed	1553	818 (19.2%)	735 (24.3%)	
	Decreased	3512	2166 (50.7%)	1346 (44.6%)	
	Would not visit others anyway	2194	1269 (29.7%)	925 (30.6%)	
Frequency of playing mahjong, chess, and cards	Increased	32	16 (0.4%)	16 (0.5%)	< 0.001
	Not Changed	485	282 (6.6%)	203 (6.7%)	
	Decreased	1362	893 (20.9%)	469 (15.5%)	
	Would not play anyway	5409	3077 (72.1%)	2332 (77.2%)	
Frequency of dancing outdoors	Increased	8	3 (0.1%)	5 (0.2%)	0.033
	Not Changed	224	113 (2.6%)	111 (3.7%)	
	Decreased	459	280 (6.6%)	179 (5.9%)	
	Would not dance outdoors anyway	6597	3872 (90.7%)	2725 (90.2%)	
Frequency of calling and messaging	Increased	1026	660 (16.7%)	366 (13.2%)	< 0.001
	Not Changed	3951	2371 (60.2%)	1580 (57.0%)	
	Decreased	1139	616 (15.6%)	523 (18.9%)	
	No device/would not do this anyway	599	296 (67.5%)	303 (110.9%)	
Frequency of internet contacting	Increased	601	437 (14.9%)	164 (8.5%)	< 0.001
	Not Changed	1446	967 (33.0%)	479 (24.8%)	
	Decreased	317	177 (6.0%)	140 (7.3%)	
	No device/would not do this anyway	2496	1349 (46.1%)	1147 (59.4%)	
Smoking	Increased	150	97 (2.3%)	53 (1.8%)	< 0.001
	Not Changed	1637	1056 (24.7%)	581 (19.2%)	
	Decreased	404	228 (5.3%)	176 (5.8%)	
	Never	5097	2887 (67.6%)	2210 (73.2%)	
Drinking	Increased	63	46 (1.1%)	17 (0.6%)	< 0.001
	Not Changed	1813	1211 (28.4%)	602 (19.9%)	
	Decreased	575	368 (8.6%)	207 (6.9%)	
	Never	4837	2643 (61.9%)	2194 (72.6%)	
Sleep duration	Increased	407	248 (5.8%)	159 (5.3%)	< 0.001
	Not Changed	6095	3672 (86.0%)	2423 (80.2%)	
	Decreased	786	348 (8.2%)	438 (14.5%)	
Food intake	Increased	138	80 (1.9%)	58 (1.9%)	< 0.001
	Not Changed	6400	3831 (89.8%)	2569 (85.1%)	
	Decreased	750	357 (8.4%)	393 (13.0%)	

Abbreviations: DS, depressive symptoms; NDS, no depressive symptoms; N, number; SD, standard deviation

### 3.2 Association of DS With Social Activities and Networking During COVID‐19

The association between depressive symptoms and specific social activities and networking during COVID‐19 is detailed in Table 2. Logistic regression models were employed to examine the associations. In model 1, factors such as time spent outdoors, time of intense activities, time of light activities, frequency of visiting others, frequency of playing mahjong, chess, cards, frequency of internet contacting, smoking, drinking, sleeping duration, and food intake were considered. Model 2 involved adjusting for demographics, including age, sex, education level, and marital status. In model 3, adjustments included demographics and mental health variables (i.e., anxiety and fears).

**Table 2:** Multiple logistic regression model testing the association between social activities during COVID‐19 and depression.

Characteristics of social activities during COVID‐19		Model 1		Model 2		Model 3	
		OR (95% CI)	*p*‐value	OR (95% CI)	*p*‐value	OR (95% CI)	*p*‐value
Times of going outdoors	Increased	1.01 (0.87, 1.14)	0.933	1.03 (0.89, 1.17)	0.688		0.942
	Not Changed	Ref		Ref		Ref	
	Decreased	0.94 (0.91, 0.96)	**< 0.001**	0.96 (0.93, 0.98)	**< 0.001**	0.92 (0.89, 0.94)	**< 0.001**
Time spent outdoors	Increased	0.96 (0.83, 1.09)	0.513	0.96 (0.83, 1.09)	0.571	0.94 (0.81, 1.07)	0.348
	Not Changed	Ref		Ref		Ref	
	Decreased	0.92 (0.89, 0.94)	**< 0.001**	0.94 (0.92, 0.96)	0.571	0.91 (0.88, 0.93)	**< 0.001**
Time of intense activities	Increased	1.10 (0.99, 1.21)	0.089	1.10 (0.98, 1.21)	0.089	1.07 (0.96, 1.18)	0.228
	Not Changed	Ref		Ref		Ref	
	Decreased	1.02 (0.99, 1.05)	0.090	1.04 (1.02, 1.07)	**0.002**	1.00 (0.98, 1.03)	0.806
Time of moderate activities	Increased	1.05 (0.99, 1.12)	0.116	1.04 (0.97, 1.10)	0.280	0.99 (0.93, 1.06)	0.865
	Not Changed	Ref		Ref		Ref	
	Decreased	0.97 (0.94, 0.99)	**0.032**	1.00 (0.97, 1.02)	0.814	0.96 (0.93, 0.99)	**0.008**
Time of light activities	Increased	1.01 (0.93, 1.08)	0.890	1.05 (0.97, 1.13)	0.248	1.02 (0.94, 1.10)	0.664
	Not Changed	Ref		Ref		Ref	
	Decreased	0.97 (0.95, 0.99)	**0.016**	0.98 (0.96, 1.01)	0.154	0.94 (0.92, 0.97)	**< 0.001**
Frequency of visiting others	Increased	1.01 (0.83, 1.19)	0.918	1.05 (0.87, 1.22)	0.624	1.00 (0.82, 1.18)	0.987
	Not Changed	Ref		Ref		Ref	
	Decreased	0.91 (0.88, 0.94)	**< 0.001**	0.94 (0.91, 0.97)	**< 0.001**	0.90 (0.87, 0.93)	**< 0.001**
	Would not visit others anyway	0.95 (0.92, 0.98)	**0.002**	0.96 (0.93, 0.99)	**0.010**	0.95 (0.92, 0.98)	**0.001**
Frequency of playing mahjong, chess, and cards	Increased	1.08 (0.91, 1.26)	0.364	1.07 (0.89, 1.25)	0.449	1.04 (0.86, 1.21)	0.675
	Not Changed	Ref		Ref		Ref	
	Decreased	0.93 (0.97, 0.98)	**0.004**	0.95 (0.90, 1.00)	0.056	0.92 (0.87, 0.97)	**0.002**
	Would not play anyway	1.01 (0.97, 1.06)	0.589	9.99 (0.94, 1.03)	0.543	0.97 (0.92, 1.02)	0.210
Frequency of dancing outdoors	Increased	1.14 (0.78, 1.48)	0.465	1.06 (0.72, 1.40)	0.750	0.98 (0.64, 1.31)	0.895
	Not Changed	Ref		Ref		Ref	
	Decreased	0.90 (0.82, 0.97)	**0.009**	0.89 (0.80, 0.96)	**0.004**	0.87 (0.78, 0.94)	**< 0.001**
	Would not dance outdoors anyway	0.92 (0.85, 0.98)	**0.014**	0.94 (0.87, 1.00)	0.060	0.94 (0.88, 1.01)	0.090
Frequency of calling and messaging	Increased	0.96 (0.92, 0.99)	**0.012**	0.98 (0.94, 1.01)	0.238	0.94 (0.91, 0.98)	**< 0.001**
	Not Changed	Ref		Ref		Ref	
	Decreased	1.06 (1.03, 1.09)	**< 0.001**	1.07 (1.03, 1.10)	**< 0.001**	1.03 (1.00, 1.06)	0.053
	No device/would not do this anyway	1.12 (1.06, 1.15)	**< 0.001**	1.06 (1.02, 1.10)	**0.005**	1.06 (1.02, 1.10)	**0.005**
Frequency of internet contacting	Increased	0.94 (0.90, 0.99)	**0.013**	0.96 (0.91, 1.00)	0.077	0.93 (0.88, 0.97)	**0.003**
	Not Changed	Ref		Ref		Ref	
	Decreased	1.12 (1.05, 1.17)	**< 0.001**	1.11 (1.05, 1.17)	**< 0.001**	1.06 (1.00, 1.12)	0.054
	No device/would not do this anyway	1.14 (1.10, 1.16)	**< 0.001**	1.07 (1.04, 1.10)	**< 0.001**	1.07 (1.03, 1.10)	**< 0.001**
Smoking	Increased	1.00 (0.92, 1.08)	0.970	1.01 (0.92, 1.09)	0.889	0.96 (0.87, 1.04)	0.292
	Not Changed	Ref		Ref		Ref	
	Decreased	1.08 (1.03, 1.13)	**0.003**	1.11 (1.05, 1.15)	**< 0.001**	1.06 (1.00, 1.11)	**0.034**
	Never	1.08 (1.05, 1.11)	**< 0.001**	0.99 (0.96, 1.02)	0.387	0.98 (0.94, 1.01)	0.110
Drinking	Increased	0.94 (0.81, 1.06)	0.322	0.96 (0.83, 1.08)	0.486	0.93 (0.80, 1.05)	0.259
	Not Changed	Ref		Ref		Ref	
	Decreased	1.03 (0.98, 1.07)	0.233	1.04 (0.99, 1.08)	0.146	1.00 (0.95, 1.04)	0.863
	Never	1.13 (1.10, 1.15)	**< 0.001**	1.05 (1.02, 1.08)	**< 0.001**	1.04 (1.01, 1.07)	**0.005**
Sleep duration	Increased	0.99 (0.94, 1.04)	0.784	1.00 (1.05, 1.10)	0.939	0.96 (0.91, 1.01)	0.161
	Not Changed	Ref		Ref		Ref	
	Decreased	1.17 (1.12, 1.20)	**< 0.001**	1.14 (1.10, 1.17)	**< 0.001**	1.07 (1.03, 1.11)	**0.001**
Food intake	Increased	1.02 (0.94, 1.11)	0.655	1.03 (0.94, 1.12)	0.503	1.01 (0.92, 1.09)	0.873
	Not Changed	Ref		Ref		Ref	
	Decreased	1.13 (1.09, 1.16)	**< 0.001**	1.12 (1.08, 1.15)	**< 0.001**	1.07 (1.03, 1.10)	**< 0.001**

*Note:* Model 1: Unadjusted. Model 2: Adjusted for social demographics (including age, sex, education level, and marital status). Model 3: Adjusted for social demographics and mental problem (including anxiety, and fears). Bold values denote statistical significance at the *p* < 0.05 level.

The analysis revealed that decreased time spent outdoors (*p* < 0.001), decreased time of intense activities (*p* < 0.001), decreased time of intense activities (*p* = 0.008), decreased frequency of visiting others (*p* < 0.001), would not visit others (*p* = 0.001), decreased frequency of playing mahjong, chess, and cards (*p* = 0.002), decreased frequency of dancing outdoors (*p* < 0.001), increased frequency of calling and messaging (*p* < 0.001), increased frequency of internet contacting (*p* = 0.003) during COVID‐19 were identified as protective factors against depressive symptoms.

Additionally, factors such as no device/would not engage in calling and messaging (*p* = 0.005), no device/would not engage in internet contacting (*p* < 0.001), decreased smoking (*p* = 0.03), never drinking (*p* = 0.005), and decreased food intake (*p* < 0.001) were identified as risk factors.

## 4 Discussion

The present study found that 41.4% of Chinese adults aged 65 and older exhibited clinically significant depressive symptoms during the COVID‐19 pandemic period assessed in 2019–2020. This alarmingly high prevalence suggests potential mental health challenges of the pandemic and associated public health restrictions in this vulnerable population. Our findings indicate that disruptions to regular social activities and habits were strongly associated with increased odds of depressive symptoms, even after controlling demographic factors and self‐reported anxiety/fear levels related to COVID‐19.

Our investigation, as outlined in Table 2, revealed noteworthy associations between specific social activities and networking behaviors and the prevalence of depressive symptoms in this demographic. Notably, the analysis identified several protective factors against depressive symptoms during the pandemic. Specifically, decreases in time spent outdoors, participation in intense and light physical activities, in‐person visiting with others, and playing games like mahjong or cards were all identified as risk factors for depression. These results align with prior evidence that lack of social engagement, sedentary behavior, and disruptions to routines and cognitively stimulating activities can precipitate or exacerbate depressive symptoms in late life [28]. From a sociological perspective, older adults often rely on these activities to maintain social bonds and a sense of community, which are critical for emotional well‐being. The restrictions imposed during the COVID‐19 pandemic, including lockdowns and physical distancing measures, likely disrupted these social networks, leading to feelings of isolation and loneliness, which are well‐documented precursors to depressive symptoms in later life [29]. Additionally, from a psychological perspective, the loss of routine and structure provided by regular outdoor or group activities may have contributed to a diminished sense of purpose or control, further exacerbating emotional distress [30, 31].

Interestingly, increased use of virtual communication through calls, messaging, and internet contact was correlated with a lower likelihood of depressive symptoms. This suggests that while in‐person social contact is important, older adults may have benefited from technology that facilitated remote social connections during lockdowns and physical distancing periods. This may be explained by the role of technology as a compensatory mechanism during periods of enforced physical isolation. Virtual interactions, while not fully replacing in‐person contact, could have provided a vital means for older adults to maintain social connections with family and friends, thereby mitigating feelings of loneliness. This finding aligns with emerging evidence that digital literacy and access to technology can play a protective role in mental health during public health crises, particularly for populations at risk of isolation [32]. Additionally, it also underscores the significance of maintaining social connections and engaging in outdoor and recreational activities, even during challenging times such as the COVID‐19 pandemic. However, the association between lack of access to or engagement with such technology and a higher likelihood of depressive symptoms suggests a potential digital divide, where those without the means or skills to use these tools may be disproportionately affected.

Certain lifestyle factors were also linked to depression risk, including not engaging in any calls/messaging or internet use, decreased smoking, never drinking alcohol, and reduced food intake during the pandemic period. The associations with smoking and alcohol use are somewhat counterintuitive, as excessive use of these substances is typically considered a risk factor for depression. However, abrupt cessation of long‐term habits could potentially contribute to psychological distress [33, 34]. For instance, older adults who reduced smoking or abstained from alcohol during the pandemic may have done so due to health concerns or financial constraints, which could introduce additional psychological stressors. Alternatively, these changes might disrupt coping mechanisms that, while unhealthy in excess, provided some emotional relief in moderation. Regarding reduced food intake, a nutritional perspective suggests that inadequate caloric or micronutrient intake could impair brain function and mood regulation through mechanisms such as reduced serotonin synthesis, which is dependent on dietary tryptophan [35]. Furthermore, reduced food intake might also reflect social isolation, as shared meals are often a key component of social interaction for older adults, and eating alone has been linked to poorer mental health outcomes [36].

From a broader epidemiological perspective, the high prevalence of depressive symptoms in this population during the COVID‐19 pandemic may also be influenced by contextual factors beyond individual behaviors. The pervasive fear of infection, economic uncertainty, and restricted access to healthcare services likely compounded psychological stress. These systemic stressors, combined with pre‐existing vulnerabilities such as chronic health conditions or limited social support, may have created a “perfect storm” for mental health challenges among older adults. While our study did not explore biological mechanisms directly, it is plausible that chronic stress from these environmental factors could activate inflammatory pathways or dysregulate the hypothalamic‐pituitary‐adrenal (HPA) axis, both of which are implicated in the pathophysiology of depression [37].

This study had several strengths, including the large, nationally representative sample, comprehensive assessment of social activities/behaviors during the pandemic lockdowns, and the ability to control for key demographic and psychological covariates. However, some limitations must be acknowledged. The cross‐sectional design precludes causal inferences about how activity changes were associated with depression. It is possible that depressive symptoms could have caused some of the observed reductions in social activity, rather than vice versa. Longitudinal follow‐up assessing changes in activities and mental health over time would strengthen causal interpretations. Additionally, the self‐report nature of data may have introduced recall or reporting biases that could not be quantified. Moreover, the study did not account for several potential confounding factors that may influence the observed association between reduced social engagement and depressive symptoms. Although adjustments were made for key demographic and psychological variables, unmeasured factors—such as pre‐existing mental health conditions, physical health status, personality traits (e.g., introversion or neuroticism), access to digital communication tools, or baseline levels of social engagement before the pandemic—could have played a significant role. These factors may confound the relationship by simultaneously affecting the likelihood of social withdrawal and the severity of depressive symptoms. Future research should incorporate these variables to isolate better the specific effects of social engagement on mental health outcomes.

In conclusion, this large study of older Chinese adults found that decreases in outdoor time, physical/social activities, and in‐person social engagement during the COVID‐19 pandemic were associated with significantly higher risks of clinically relevant depressive symptoms. Conversely, maintenance of virtual social connections appeared protective. Certain unfavorable lifestyle changes, including reduced smoking/drinking cessation and inadequate nutrition, were also implicated as potential risk factors. As nations continue responding to the evolving COVID‐19 situation, these findings underscore the importance of promoting social participation, physical activity, cognitive stimulation, and healthy behaviors among vulnerable older populations to mitigate adverse mental health consequences. Community‐based programs, technology‐facilitated interventions, and policy efforts supporting high‐risk groups may be warranted to preserve cognitive, mental, and social wellbeing during public health emergencies and their aftermath.

## Author Contributions

**Zheng Guo:** conceptualization, writing – review and editing, writing – original draft, formal analysis, validation, methodology, software. **Yulu Zheng:** software, data curation, writing – review and editing, writing – original draft, visualization, formal analysis. **Yahong Zhu:** investigation, writing – review and editing, validation, software, data curation, resources. **Huacheng Wang:** resources, data curation, project administration, visualization, writing – review and editing. **Jian Geng:** writing – review and editing, validation, project administration, resources. **Hengrui Liu:** validation, writing – review and editing, methodology, software, resources. **Piyong Ma:** methodology, data curation, writing – review and editing, investigation. **Lois Balmer:** resources, supervision, writing – review and editing, conceptualization. **Xingang Li:** writing – review and editing, writing – original draft, funding acquisition, conceptualization, supervision.

## Disclosure

All authors have read and approved the final version of the manuscript. Xingang Li and Lois Balmer have full access to all of the data in this study and take complete responsibility for the integrity of the data and the accuracy of the data analysis.

## Ethics Statement

The original CHARLS obtained approval from the Biomedical Ethics Review Committee of Peking University (IRB00001052‐11015) and was conducted in compliance with the Declaration of Helsinki (revised in Edinburgh 2000). All participants provided signed and informed consent before their involvement in the study. The current study was exempted for taking ethical approval and informed consent by the Ethics review committee of Edith Cowan University (Registration No. 2025‐06753‐LI).

## Conflicts of Interest

The authors declare no conflicts of interest.

## Transparency Statement

The lead authors Lois Balmer and Xingang Li affirm that this manuscript is an honest, accurate, and transparent account of the study being reported; that no important aspects of the study have been omitted; and that any discrepancies from the study as planned (and, if relevant, registered) have been explained.

## Data Availability

The datasets analyzed are open access to request online at charls.pku.edu.cn/.

## References

[1] A. J. Ferrari , D. F. Santomauro , A. Aali , et al., “Global Incidence, Prevalence, Years Lived With Disability (YLDs), Disability‐Adjusted Life‐Years (DALYs), and Healthy Life Expectancy (HALE) for 371 Diseases and Injuries in 204 Countries and Territories and 811 Subnational Locations, 1990–2021: A Systematic Analysis for the Global Burden of Disease Study 2021,” Lancet 403, no. 10440 (2024): 2133–2161, 10.1016/s0140-6736(24)00757-8. 38642570 PMC11122111

[2] E. R. Walker , R. E. McGee , and B. G. Druss , “Mortality in Mental Disorders and Global Disease Burden Implications: A Systematic Review and Meta‐Analysis,” JAMA Psychiatry 72, no. 4 (2015): 334–341, 10.1001/jamapsychiatry.2014.2502. 25671328 PMC4461039

[3] V. Patel , D. Chisholm , R. Parikh , et al., “Addressing the Burden of Mental, Neurological, and Substance Use Disorders: Key Messages From Disease Control Priorities, 3rd edition,” Lancet 387, no. 10028 (2016): 1672–1685, 10.1016/s0140-6736(15)00390-6. 26454360

[4] C. K. Ettman , G. H. Cohen , S. M. Abdalla , et al., “Persistent Depressive Symptoms During COVID‐19: A National, Population‐Representative, Longitudinal Study of U.S. Adults,” Lancet Regional Health Americas 5 (2022): 100091, 10.1016/j.lana.2021.100091. 34635882 PMC8488314

[5] J. Hwang , S. Park , and S. Kim , “Effects of Participation in Social Activities on Cognitive Function Among Middle‐Aged and Older Adults in Korea,” International Journal of Environmental Research and Public Health 15, no. 10 (2018): 2315, 10.3390/ijerph15102315. 30347887 PMC6210154

[6] S. H. Lee and Y. B. Kim , “Which Type of Social Activities May Reduce Cognitive Decline in the Elderly?: A Longitudinal Population‐Based Study,” BMC Geriatrics 16, no. 1 (2016): 165, 10.1186/s12877-016-0343-x. 27677321 PMC5039914

[7] Z. Fan , X. Lv , L. Tu , M. Zhang , X. Yu , and H. Wang , “Reduced Social Activities and Networks, but Not Social Support, Are Associated With Cognitive Decline Among Older Chinese Adults: A Prospective Study,” Social Science & Medicine 289 (2021): 114423, 10.1016/j.socscimed.2021.114423. 34597879

[8] J. Kim and S. Hwang , “Separating the Effects of Transitions Into and Out of Social Isolation and Loneliness on Cognitive Function in Later Life,” Journals of Gerontology. Series B, Psychological Sciences and Social Sciences 79, no. 7 (2024): gbae082, 10.1093/geronb/gbae082. 38742600

[9] J. Kim and G.‐R. Park , “Cumulative Exposure to Social Isolation and Longitudinal Changes in Life Satisfaction Among Older Adults,” Society and Mental Health 14, no. 2 (2024): 113–128.

[10] J. Kim and G. R. Park , “Prolonged Social Isolation and Cognitive Function in Older Adults: Lack of Informal Social Contact Versus Formal Social Activity as the Source of Social Isolation,” Aging & Mental Health 27, no. 12 (2023): 2438–2445, 10.1080/13607863.2023.2202616. 37079761

[11] Z. Guo , R. Meng , Y. Zheng , et al., “Translation and Cross‐Cultural Validation of a Precision Health Tool, the Suboptimal Health Status Questionnaire‐25, in Korean,” Journal of Global Health 12 (2022): 04077, 10.7189/jogh.12.04077. 36181723 PMC9526479

[12] C. Ozemek , C. J. Lavie , and Ø. Rognmo , “Global Physical Activity Levels ‐ Need for Intervention,” Progress in Cardiovascular Diseases 62, no. 2 (2019): 102–107, 10.1016/j.pcad.2019.02.004. 30802461

[13] T. Wei , Z. Guo , Z. Wang , et al., “Five Major Psychiatric Disorders and Alzheimer's Disease: A Bidirectional Mendelian Randomization Study,” Journal of Alzheimer's Disease 87, no. 2 (2022): 675–684, 10.3233/jad-220010. 35367968

[14] M. Nicola , Z. Alsafi , C. Sohrabi , et al., “The Socio‐Economic Implications of the Coronavirus Pandemic (COVID‐19): A Review,” International Journal of Surgery 78 (2020): 185–193, 10.1016/j.ijsu.2020.04.018. 32305533 PMC7162753

[15] H. Wang , Z. Guo , Y. Zheng , and B. Chen , “Genetic Liability Between COVID‐19 and Heart Failure: Evidence From a Bidirectional Mendelian Randomization Study,” BMC Cardiovascular Disorders 22, no. 1 (2022): 262, 10.1186/s12872-022-02702-w. 35690714 PMC9188011

[16] D. Schröder , C. Müllenmeister , S. Heinemann , et al., “Social Participation During the COVID‐19 Pandemic In Persons With a High Risk for a Severe Course of COVID‐19—Results of a Longitudinal, Multi‐Center Observational Study in Germany,” Health Psychology and Behavioral Medicine 11, no. 1 (2023): 2249534, 10.1080/21642850.2023.2249534. 37645515 PMC10461510

[17] T. Noguchi , T. Hayashi , Y. Kubo , N. Tomiyama , A. Ochi , and H. Hayashi , “Association Between Decreased Social Participation and Depressive Symptom Onset Among Community‐Dwelling Older Adults: A Longitudinal Study During the COVID‐19 Pandemic,” Journal of Nutrition, Health & Aging 25, no. 9 (2021): 1070–1075, 10.1007/s12603-021-1674-7. PMC8440728 34725663

[18] C. Karing , “Prevalence and Predictors of Anxiety, Depression and Stress Among University Students During the Period of the First Lockdown in Germany,” Journal of Affective Disorders Reports 5 (2021): 100174, 10.1016/j.jadr.2021.100174. 34642682 PMC8497174

[19] H. Dietrich , A. Patzina , and A. Lerche , “Social Inequality in the Homeschooling Efforts of German High School Students During a School Closing Period,” supplement, European Societies 23, no. S1 (2021): S348–S369.

[20] L. Schmid , J. Wörn , K. Hank , B. Sawatzki , and S. Walper , “Changes in Employment and Relationship Satisfaction in Times of the COVID‐19 Pandemic: Evidence From the German Family Panel,” supplement, European Societies 23, no. S1 (2021): S743–S758.

[21] H. Hou , X. Feng , Y. Li , et al., “Suboptimal Health Status and Psychological Symptoms Among Chinese College Students: A Perspective of Predictive, Preventive and Personalised Health,” EPMA Journal 9, no. 4 (2018): 367–377, 10.1007/s13167-018-0148-4. 30538788 PMC6261912

[22] M. L. Frutos , D. P. Cruzado , D. Lunsford , S. G. Orza , and R. Cantero‐Téllez , “Impact of Social Isolation Due to COVID‐19 on Daily Life Activities and Independence of People over 65: A Cross‐Sectional Study,” International Journal of Environmental Research and Public Health 20, no. 5 (2023): 4177, 10.3390/ijerph20054177. 36901189 PMC10001756

[23] Y. Zhao , Y. Hu , J. P. Smith , J. Strauss , and G. Yang , “Cohort Profile: The China Health and Retirement Longitudinal Study (CHARLS),” International Journal of Epidemiology 43, no. 1 (2014): 61–68, 10.1093/ije/dys203. 23243115 PMC3937970

[24] M. Irwin , K. H. Artin , and M. N. Oxman , “Screening for Depression in the Older Adult: Criterion Validity of the 10‐item Center for Epidemiological Studies Depression Scale (CES‐D),” Archives of Internal Medicine 159, no. 15 (1999): 1701–1704, 10.1001/archinte.159.15.1701. 10448771

[25] H. Chen and A. C. Mui , “Factorial Validity of the Center for Epidemiologic Studies Depression Scale Short Form in Older Population in China,” International Psychogeriatrics 26, no. 1 (2014): 49–57, 10.1017/s1041610213001701. 24125553

[26] E. M. Andresen , J. A. Malmgren , W. B. Carter , and D. L. Patrick , “Screening for Depression in Well Older Adults: Evaluation of a Short Form of the CES‐D,” American Journal of Preventive Medicine 10, no. 2 (1994): 77–84. 8037935

[27] W. Lin , “A Study on the Factors Influencing the Community Participation of Older Adults in China: Based on the CHARLS2011 Data Set,” Health & Social Care in the Community 25, no. 3 (2017): 1160–1168, 10.1111/hsc.12415. 28178751

[28] Z. Feng , Q. Li , L. Zhou , Z. Chen , and W. Yin , “The Relationship Between Depressive Symptoms and Activity of Daily Living Disability Among the Elderly: Results From the China Health and Retirement Longitudinal Study (CHARLS),” Public Health 198 (2021): 75–81, 10.1016/j.puhe.2021.06.023. 34365109

[29] J. T. Cacioppo , L. C. Hawkley , and R. A. Thisted , “Perceived Social Isolation Makes Me Sad: 5‐year Cross‐Lagged Analyses of Loneliness and Depressive Symptomatology in the Chicago Health, Aging, and Social Relations Study,” Psychology and Aging 25, no. 2 (2010): 453–463, 10.1037/a0017216. 20545429 PMC2922929

[30] M. N. Hossain , J. Lee , H. Choi , Y. S. Kwak , and J. Kim , “The Impact of Exercise on Depression: How Moving Makes Your Brain and Body Feel Better,” Physical Activity and Nutrition 28, no. 2 (2024): 43–51, 10.20463/pan.2024.0015. PMC11298280 39097997

[31] Y. Takiguchi , M. Matsui , M. Kikutani , and K. Ebina , “The Relationship Between Leisure Activities and Mental Health: The Impact of Resilience and COVID‐19,” Applied Psychology: Health and Well‐Being 15, no. 1 (2023): 133–151, 10.1111/aphw.12394. 35971651 PMC9538683

[32] B. A. Smith , A. M. Georgiopoulos , A. Mueller , et al., “Impact of COVID‐19 on Mental Health: Effects on Screening, Care Delivery, and People With Cystic Fibrosis,” supplement, Journal of Cystic Fibrosis 20, no. S3 (2021): 31–38, 10.1016/j.jcf.2021.08.027. PMC8716152 34930540

[33] B. Gibson , B. A. Rosser , J. Schneider , and M. J. Forshaw , “The Role of Uncertainty Intolerance in Adjusting to Long‐Term Physical Health Conditions: A Systematic Review,” PLoS One 18, no. 6 (2023): e0286198, 10.1371/journal.pone.0286198. 37267292 PMC10237456

[34] D. P. Fernandez , D. J. Kuss , and M. D. Griffiths , “Short‐Term Abstinence Effects Across Potential Behavioral Addictions: A Systematic Review,” Clinical Psychology Review 76 (2020): 101828, 10.1016/j.cpr.2020.101828. 32062303

[35] K. S. Young , “Cognitive Behavior Therapy With Internet Addicts: Treatment Outcomes and Implications,” Cyberpsychology & Behavior 10, no. 5 (2007): 671–679, 10.1089/cpb.2007.9971. 17927535

[36] Y. Tani , Y. Sasaki , M. Haseda , K. Kondo , and N. Kondo , “Eating Alone and Depression in Older Men and Women by Cohabitation Status: The JAGES Longitudinal Survey,” Age and Ageing 44, no. 6 (2015): 1019–1026. 26504120 10.1093/ageing/afv145 PMC4621239

[37] A. H. Miller and C. L. Raison , “The Role of Inflammation in Depression: From Evolutionary Imperative to Modern Treatment Target,” Nature Reviews Immunology 16, no. 1 (2016): 22–34, 10.1038/nri.2015.5. PMC5542678 26711676

